# Analysis of the effectiveness of a new system of automatic spray hands in ICU

**DOI:** 10.1186/2197-425X-3-S1-A1017

**Published:** 2015-10-01

**Authors:** J Canovas, A Lopez, D Torres, A Burruezo, L Capilla, A Carrillo

**Affiliations:** Hospital Morales Meseguer, ICU, Murcia, Spain

## Introduction

Hand hygiene is the most important method to prevent the spread of infections, according to the Center for Disease Control and Prevention (CDC) in the United States. Therefore, we designed a pilot study to assess the antisepsis of the hands through the use of a new electronic device for automatic spray with a water-alcohol solution.

## Objetive

Analyze the effectiveness of the sterilization hands of the medical staff in an Intensive Care Unit by spraying an hydroalcoholic solution with an electronic device

## Methods

The study was carried out on voluntary optional staff of the Intensive Care Unit of our hospital, randomized, without prior notice and without any special antisepsis in the hour prior to sampling. The dispenser has a steel chamber with a photoelectric cell that detects the introduction of the hands and activates its operation by spraying the hydroalcoholic solution.

In the study used Sterillium^®^ solution (Laboratories Hartmann) as antiseptic agent, dispensing 3 milliliters per application in a time of 3 seconds. The samples were taken using sterile swabs soaked in thioglycollate over the surface of the hands of the candidate, both in the palm as in the back and with special emphasis in the interdigital spaces, and the fingernail bed. Subsequently, the swab was immersed in a known volume of thioglycolate and stirred for 30 seconds. Then, the hand hygiene dispenser was made with automatic according to protocol, and dry the product by friction for 30 seconds and repeated the sampling operation. Once in the microbiology laboratory, the sample tubes were mixed up and proceeded to the planting of 100 microliters blood agar plates (Biomerieux^®^). Plates were sealed with Parafilm and incubated aerobically at 37 ° C and at a concentration of 5% CO2 for 48 hours. Subsequently proceeded to recount the CFU (Colony-forming units) on the samples before and after hand hygiene for each case. After the CFU obtained sowing depending on volume, we calculated the reduction factor of growth obtained to evaluate the effectiveness of the procedure.

## Results

Were taken 40 samples (20 before and 20 after hand antisepsis). In the previous 20 samples obtained microbial growth, becoming one of the countless cases and ranged between 2.18 and 4.56 the logarithm of CFU. In subsequent samples of antisepsis, growth was observed only in 2 cases and logarithm was between 1.7 and 2. The reduction factor could be calculated in 19 cases being found between 1.18 and 4.56, so the average was 3.24 ± 0.76.

## Conclusions

The use according to protocol of hydroalcoholic solution sprayed with this new device significantly reduced the transitory flora in all the cases studied, having been canceled their full growth in 90% of them. The dispensing through this new electronic device could help to improve compliance with hand hygiene.

